# Building an interdisciplinary team set on bringing the sense of smell to computers

**DOI:** 10.1016/j.isci.2021.102136

**Published:** 2021-02-16

**Authors:** Alexander B. Wiltschko

**Affiliations:** 1 Google Research, Cambridge, MA, USA

**Figure:**
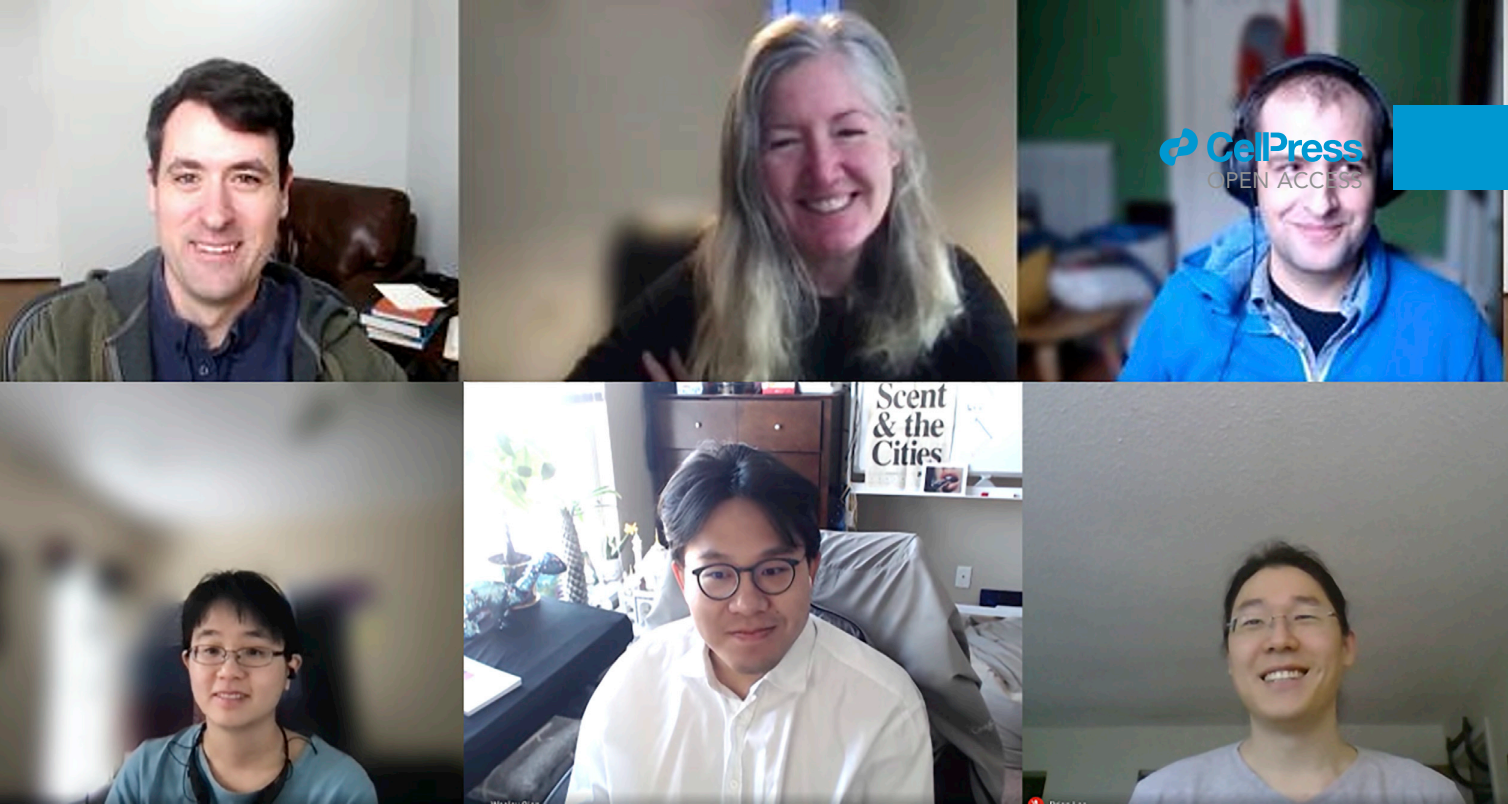
Dr. Wiltschko and his team, like the rest of the world, have had to turn to the virtual world this past year. However, when your work is so focused on how things smell, sometimes this can be challenging. The team here smiles for a “selfie” in one of their many virtual calls. (Top Row, left to right: Alexander B. Wiltschko, Kathleen Nix, Benjamin Sanchez-Lengeling; Bottom Row, left to right: Jennifer Wei, Wesley Wei Qian, Brian K. Lee)

Have you ever thought about how something could possibly smell without a nose? Dr. Alexander Wiltschko leads a team at Google Brain that aims to give computers a sense of smell. They have observed that all breakthroughs in artificial intelligence (AI) ultimately tie back to the limited sensory abilities of computers. For example, computers can see (cameras), hear (microphones), and touch (keyboards, mice, and touchscreens). Because of digital cameras, we have image data sets, and because of those data sets, we have the deep learning revolution. The story is similar for microphones, which give computers the ability to hear, which unlocks text-to-speech and speech-to-text AI applications. Computers have a sense of touch via keyboards and touchscreens, which gives rise to the entire internet, and AI language models similar to those underpinning autocomplete or Google Search.

*Each new sensory modality created a whole field of AI*. So, if there was a way scientists could give computers the sense of smell, a whole new sensory modality, it would spawn a new field of science. This is the interdisciplinary problem where Dr. Wiltschko's team is focusing their efforts currently. The way his team was assembled, along with the challenges (and possibilities) of working in an interdisciplinary team in industry, is the highlight of this backstory, as well an overarching theme of the special issue entitled “Machine Learning for Molecules and Materials” by *iScience* and *Trends in Chemistry* (https://www.sciencedirect.com/journal/iscience/special-issue/10M39TQV6KG) which Dr. Wiltschko was a guest editor.

## Proximity

### Who were the players in this project, and how did you bring everyone together?

Early on, it was clear we needed an interdisciplinary team with expertise in bench chemistry (to recognize whether molecules are safe or harmful, and easy or hard to make), computational chemistry (to model the behavior of molecules), machine learning (to make novel and highly accurate predictions on chemical data), and machine learning interpretability (to explain *why* our models make the predictions they do). We grew the team slowly, starting first with core technical skills, and focused on a narrow scientific challenge for over a year — can we predict what a molecule smells like? Eventually, as we took on more work and harder challenges, we came to need non-technical roles like program management to keep multiple interlocking projects and partners on track), and business negotiation (to gain access to new data and problems).

“Early on, it was clear we needed an interdisciplinary team…”

## Language

### Did you encounter any challenges or any benefits of working with people from different backgrounds and expertise?

The pandemic shattered all our initial plans, and how our project was progressing. Because part of our research requires people to smell jars of physical material, we had to rewrite our approach to be completely virtual. As a result, we spend a lot of ‘virtual’ time in cross-functional teams, drawing on very different expertises and perspectives. As such, a lot of my energy is devoted to ‘translation’ from one discipline to another. Everyone involved in a multi-disciplinary project must take on some of this work, but as project lead, I take on the brunt of the work. There is a constant communication overhead when working with diverse skillsets, but this tax is definitely worth it, because it allows you to solve harder, more complex problems. A book that has inspired a lot of how I think about building and running diverse teams working on a complex problem is “Team of Teams” by General Stanley McChrystal.

## Research methods

### Did this project require tailoring your research methods to adjust to working interdisciplinarity?

Our methods ‘toolkit’ includes the machine learning models that have broad use in pharmaceutical and biotechnology applications. With a solid background in machine learning, the chemistry-specific methods require a learning curve to pick up but luckily do not require a deep re-education. Because our team is currently small, we have hired people with skills in machine learning for chemistry thus avoiding the need to train the technical team members too deeply. However, the required quality of engineering at Google is extremely high, and former academics (myself included) do take some time to learn how to write cleaner and well-tested code.

We invent new methods when it is necessary, but we do our best to borrow the state-of-the-art from the literature (be it in odor psychophysics, graph neural networks, or even optical character recognition). By putting the problem first, the methods that we need become clear over time.

“By putting the problem first, the methods that we need become clear over time.”

## Governance

### How did the decision of branching out from your fields come about?

Currently, the sense of smell is unfortunately an ‘intellectual backwater’ — compared to vision and hearing, it is *much* less studied. You could view this as a challenge, or you could view this as an incredible opportunity. It is obviously very risky to embark out on a journey into an area that much of science has ignored, but for me, the thrill of exploring new territory is worth it. At Google, we have one funder — Google. So, as long as the company views this as an opportunity which will make machines more intelligent and improve people's lives, we have the ability to continue our research.

I view giving computers a new sensory modality as one of the highest leverage activities in AI today. Without cameras, there would be no computer vision algorithms, and thus no Google Image Search. The same is true for microphones, accelerometers, keyboards, and touch screens. If we gave computers a sense of smell, the possibilities are quite grand, and this is exciting both at the company level and at a personal level, which is a nice position to be at in terms of thinking about a career.

For maintaining and furthering our careers, we publish, we give external talks, and otherwise operate like an academic laboratory. It is not a given that we will succeed in creating computers that can smell, which is why we think of ourselves as a research team with all of our results meaning something (even the negative ones) — we are trying to de-risk the opportunity. So although the science itself is risky (because our goals are so ambitious!), we mitigate its impact on our careers by publishing what we find and engaging with the external community.

## Final thoughts

### What did you learn about interdisciplinary research from the project and what tips would you give to anyone considering undertaking such work?

First — engaging in interdisciplinary research and building interdisciplinary teams are both enormous challenges, because there is a ‘communication overhead’ that you cannot get rid of or ignore. Experts in chemistry have to learn to talk to experts in contract negotiation who have to learn to talk to software engineers. Learning to speak multiple professional dialects is time-consuming and sometimes exhausting. This extra work to translate your thoughts into words that are comprehensible by someone with a very different background than you can make the project feel like it is moving slowly. It certainly feels that if everybody were just focused on their narrow domain, their own micro-discipline, progress would be easier. However, blending many peoples' skillsets is incredibly rewarding because bringing together a diverse group of smart and highly motivated people can turn what might seem like an impossible problem into an incredible opportunity.

